# Longitudinal Assessment of Nasopharyngeal Biomarkers Post-COVID-19: Unveiling Persistent Markers and Severity Correlations

**DOI:** 10.1021/acs.jproteome.4c00536

**Published:** 2024-10-11

**Authors:** Francisco Javier Redondo-Calvo, Yoana Rabanal-Ruiz, Gema Verdugo-Moreno, Natalia Bejarano-Ramírez, Raquel Bodoque-Villar, Mario Durán-Prado, Soledad Illescas, Eduardo Chicano-Galvez, Francisco Javier Gómez-Romero, José Martinez-Alarcón, Javier Arias-Pardilla, Pilar Lopez-Juarez, Juan Fernando Padin, Juan Ramón Peinado, Leticia Serrano-Oviedo

**Affiliations:** † Department of Anesthesiology and Critical Care Medicine, University General Hospital, SESCAM, Ciudad Real 13004, Spain; ‡ Traslational Investigation Unit, University General Hospital, SESCAM. Research Institute of Castilla-La Mancha (IDISCAM), Ciudad Real 13004, Spain; § Faculty of Medicine, University of Castilla-La Mancha, Castilla La Mancha, Ciudad Real 13071, Spain; ∥ Oxidative Stress and Neurodegeneration Group, Medical Sciences Department, Medical School, UCLM, Regional Centre for Biomedical Research, Research Institute of Castilla-La Mancha (IDISCAM), University of Castilla-La Mancha, Ciudad Real 13071, Spain; ¶ Department of Pediatrics, University General Hospital, Ciudad Real 13004, Spain; ∇ Department of Microbiology, University General Hospital, Ciudad Real 13004, Spain; ○ Department of Medical Sciences, School of Medicine at Ciudad Real, University of Castilla-La Mancha, Ciudad Real 13071, Spain; ⧫ IMIBIC Mass Spectrometry and Molecular Imaging Unit (IMSMI). Maimonides Biomedical Research Institute of Cordoba (IMIBIC), Reina Sofia University Hospital, University of Cordoba (UCO), Córdoba 14004, Spain

**Keywords:** COVID-19, proteomics, SARS-CoV-19, biomarkers

## Abstract

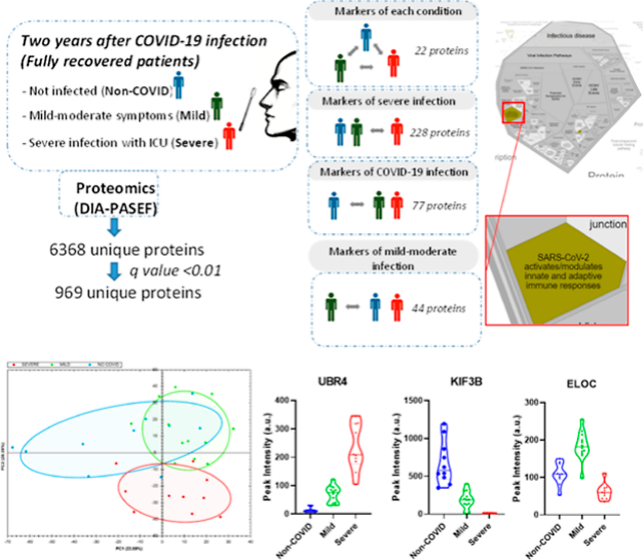

SARS-CoV-19 infection provokes a variety of symptoms; most patients present mild/moderate symptoms, whereas a small proportion of patients progress to severe illness with multiorgan failure accompanied by metabolic disturbances requiring ICU-level care. Given the importance of the disease, researchers focused on identifying severity-associated biomarkers in infected patients as well as markers associated with patients suffering long-COVID. However, little is known about the presence of biomarkers that remain a few years after SARS-CoV-2 infection once the patients fully recover of the symptoms. In this study, we evaluated the presence of persistent biomarkers in the nasopharyngeal tract two years after SARS-Cov-2 infection in fully asymptomatic patients, taking into account the severity of their infection (mild/moderate and severe infections). In addition to the direct identification of several components of the Coronavirus Infection Pathway in those individuals that suffered severe infections, we describe herein 371 proteins and their associated canonical pathways that define the different adverse effects of SARS-CoV-2 infections. The persistence of these biomarkers for up to two years after infection, along with their ability to distinguish the severity of the infection endured, highlights the surprising presence of persistent nasopharyngeal exudate changes in fully recovered patients.

## Introduction

Since the onset of the global COVID-19 pandemic, caused by the severe acute respiratory syndrome coronavirus (SARS-CoV-2), extensive research efforts have been directed toward understanding molecular mechanisms underlying the infection of the virus in order to develop strategies to face the disease.^1^ The main problem that researchers find is based on the fact that COVID-19 is a multifaceted disease characterized by a wide range of clinical manifestations, spanning from asymptomatic cases to severe clinical courses.^2^ The majority of COVID-19 patients present with asymptomatic or mild/moderate symptoms, while others experience clinical manifestations that lead to acute lung injury, systemic inflammatory response syndrome, acute respiratory distress syndrome, and multiorgan failure that require admission to intensive care units (ICUs).^3−5^

Several proteomics studies have been conducted on COVID-19 patients at the moment of infection, thus allowing a better understanding of several molecular aspects of the infection pathways. These experiments have been carried out using mainly blood plasma but also urine, biopsy samples, and nasopharyngeal exudates.^6−9^ Now that over two years have passed since the onset of the pandemic, there are additional studies in search of biomarkers of those individuals who suffer the so-called long-COVID, as they continue to experience persistent or new symptoms that broadly impact quality of life and functional status long after the initial infection has been resolved.^10−12^ These proteomic studies, mostly carried out in blood samples of patients, propose that the mechanisms reside in immunological and inflammatory dysfunction, endothelial dysfunction, and metabolic and clotting abnormalities (reviewed by ref (13)). Notwithstanding this, no study has explored biomarkers obtained from survivors after complete resolution of mild or severe physiological conditions. Given that SARS-CoV-2 primarily spreads via respiratory droplets, with the respiratory tract serving as the primary gateway for host entry, investigating the proteins within nasopharyngeal exudate samples will shed light on the persistent invisible consequences of the disease and help identify pre-existing factors that may have contributed to the severity of the infection.

## Material and Methods

### Population

Biological samples were analyzed from people diagnosed with COVID-19 or those who had been close contacts of patients diagnosed with COVID-19 between January 2021 and September 2021, whose reference healthcare setting was the Integrated Healthcare Service of Ciudad Real. Three differential groups were established: patients with COVID-19 requiring admission to an intensive care unit (ICU), COVID-19 patients with mild/moderate symptomatology with or without hospital admission, and asymptomatic persons who tested negative for COVID-19 by PCR, transcription-mediated amplification (TMA), or antigenic test, being cohabitants/close contacts of patients diagnosed with COVID-19. The study was approved by the Ethics Committee for Research with Medicines of the Integrated Service of Ciudad Real (Act no. 05/2022). Code C-526.

### Inclusion Criteria

Patients were diagnosed with COVID-19 prior to September 2021 with positive PCR, TMA, and/or antigenic test. Asymptomatic individuals without positive PCR and/or antigenic test who have been close contacts with patients diagnosed prior to September 2021. People over 18 years of age and male or female gender.

### Exclusion Criteria

Patients had any type of respiratory infection at the time of sampling. Patients with a clinical history of allergic asthma or pulmonary atopy reactions or chronic obstructive pulmonary disease/administration of inhalation medication or with the potential to interact with the respiratory system. Patients who did not submit signed documents or whose informed consent was not obtained at a date prior to the study or any specific intervention.

### Sample Acquisition

A search was carried out through the management control and analytical accounting service for the minimum basic data set (CMBD) of the Integrated Healthcare Service of Ciudad Real of patients with the criteria indicated in the protocol for the three established study groups, which was subsequently contrasted by the Microbiology Service. The patients were selected randomly from these lists. Clinical practitioners from the research team contacted the patients to inform them of the study and sign the Informed Consent form.

The patients were summoned to the University General Hospital to obtain two samples of nasopharyngeal exudate using the usual swab technique. One sample was used for proteomics and the other for multiplex PCR to rule out coinfections. The management of the sample was processed through the Tissue Biobank of the General University Hospital of Ciudad Real.

### Sample Screening

Respiratory virus infection was ruled out using a qualitative multiplex PCR (Biofire Respiratory Panel 2.1, bioMérieux) in nasopharyngeal samples collected with a flocked swab in virus transport medium (UTM, Copan) following the manufacturer’s instructions. This technique allows detection of the following viruses: adenovirus, coronavirus 229E, HKU1, NL63, OC43, and SARS-CoV-2, metapneumovirus, rhinovirus/enterovirus, influenza A and B, parainfluenza 1, 2, 3, and 4, and respiratory syncytial virus, in addition to the following bacteria: *Bordetella pertussis* and parapertussis, *Chlamydia pneumoniae*, and *Mycoplasma pneumoniae*. Furthermore, we searched for COVID proteins (https://www.ncbi.nlm.nih.gov/datasets/taxonomy/2697049/) within the data set of the proteomics results in order to find any possible presence of the virus. Neither DIA-NN (https://github.com/vdemichev/DiaNN/releases/tag/1.8.1) nor Spectronaut were able to detect COVID proteins in the samples, which corroborated the previous microbiological study.

### Cohort Description

35 samples were collected from the people who met the inclusion criteria. The multiplex PCR results were negative for all patients except for three patients who were ruled out of the study due to respiratory symptoms and positivity for virus, two of them for rhinovirus/enterovirus and one for coronavirus 229E. Once these samples were discarded, the study was carried out on 32 samples distributed into three groups: severe, for patients who required admission to the ICU (*n* = 10); mild, for patients with moderate or mild symptoms with or without hospital admission (*n* = 12); and non-COVID, for people with a negative diagnosis of the disease (*n* = 10) (Table 1). We found no statistically significant differences between the study groups in relation to demographic characteristics and comorbidities.

**Table 1:** Demographic and Clinical Characteristics of the COVID-19 Cohort and Controls^a^

characteristics	controls (*n* = 10)	mild-moderate (*n* = 12)	severe (*n* = 10)	*p*-value
Gender [*n* (%)]				
male	8 (80)	5 (42)	7 (70)	0.152
female	2 (20)	7 (58)	3 (30)	
Age (years) [mean ± SEM]	47.80 ± 3.24	53.75 ± 3.77	58.90 ± 3.52	0.121^b^
Vaccine* [*n* (%)]				
post	8 (100)	12 (100)	9 (90)	0.355
Comorbidity [*n* (%)]				
hypercholesterolemia	1 (10)	2 (17)	2 (20)	0.821
hypothyroidism	0 (0)	0 (0)	1 (10)	0.321
overweight	0 (0)	0 (0)	2 (20)	0.096
dyslipidemia	1 (10)	1 (8)	2 (20)	0.683
high blood pressure	0 (0)	3 (25)	4 (40)	0.091
arthrosis	0 (0)	0 (0)	1 (10)	0.321
acute myocardial infarction	0 (0)	2 (17)	0 (0)	0.169
smoker	1 (10)	1 (8)	0 (0)	0.608
Symptoms [*n* (%)]				
fever	0 (0)	3 (25)	7 (70)	N/A
gastrointestinal	0 (0)	1 (8)	0 (0)	N/A
musculoskeletal	0 (0)	1 (8)	0 (0)	N/A
sensory	0 (0)	1 (8)	0 (0)	N/A
dizziness	0 (0)	1 (8)	0 (0)	N/A
cough	0 (0)	7 (58)	5 (50)	N/A
anosmia	0 (0)	3 (25)	0 (0)	N/A
dyspnea	0 (0)	0 (0)	7 (70)	N/A
weight loss	0 (0)	1 (8)	0 (0)	N/A
asthenia	0 (0)	3 (25)	1 (10)	N/A
coma	0 (0)	0 (0)	1 (10)	N/A
epistaxis	0 (0)	0 (0)	1 (10)	N/A
cold	1 (10)	2 (17)	1 (10)	N/A
amnesia	0 (0)	0 (0)	1 (10)	N/A
headache	0 (0)	2 (17)	1 (10)	N/A
Treatments [*n* (%)]				
antibiotics	0 (0)	1 (8)	10 (100)	N/A
heparin	0 (0)	0 (0)	10 (100)	N/A
oxygen	0 (0)	5 (42)	10 (100)	N/A
corticoids	0 (0)	3 (25)	10 (100)	N/A
aerosol/inhalator	0 (0)	1 (8)	10 (100)	N/A

a Data are *n* (%), mean ± standard error of the mean (SEM). NA = not applicable. *The vaccination data shown are for those vaccinated after direct contact in the control group or after infection in the mild or severe group. *P*-value (X2 Pearson, ANOVA).

b ANOVA.

All patients in the severe group required more treatment, developed more severe symptomatology, and were admitted to the ICU for an average of 29.0 ± 9.31 days and required invasive ventilation. In the mild COVID-19 group, three of the patients were admitted to the hospital for less than 1 week. Quantitative variables were analyzed using the mean percentage and SEM. Qualitative variables were expressed as counts and frequencies, *n* (%). Statistical analysis was conducted using SPSS 29.0 (IBM SPSS, Armonk, NY, USA).

### Sample Preparation and Protein Quantification

Samples were resuspended in 500 μL of PBS supplemented with protease inhibitors (1183617001; Roche) on ice, and protein concentrations were measured using a bicinchoninic acid protein assay (786–570; GB Biosciences). Proteins were precipitated using a dual-phase MeOH/CHCl_3_/H_2_O extraction, and dry samples were stored at −80 °C until analysis.

### Experimental Procedure

Samples were resuspended in the RapiGest surfactant (Waters, UK), followed by total protein amount quantification for each sample (Qubit Protein Assay). Then, samples were manually digested with advanced iST (in-stage-tip) double digestion using Trypsin and LysC as enzymes. The digested samples were then resuspended in a phase A solution (LC–MS water solution containing 0.1% formic acid) and subsequently diluted to a stock solution of 20 ng/μL. A pool of all samples was created by mixing the same volume for each individual sample for internal experiment quality control (QC) purposes. Both samples and the QC pool were analyzed by LC–MS/MS using the DIA-PASEF mode on the EvosepOne-TIMSTOF-Flex Clinical Proteomics Platform available at the IMSMI Unit. During acquisition, the samples were monitored and identified in real time by PaSER software (https://cutt.ly/L0hMYde) to ensure the correct data acquisition (QC). To avoid batch effects, samples were acquired in a randomized order. Additionally, a QC sample analysis was performed using the above-indicated QC pool in order to control system stability during DIA-PASEF acquisition. In addition to the pool QC, some of the samples were run in duplicate to ensure intrasample reproducibility.

### LC–MS

To perform the chromatography and MS analysis, a Bruker PepSep C18 column was used (8 cm × 150 μm, 1.5 μm, part no. 1893470) with an EvosepOne nanoLC (Evosep, Odense, Denmark, 2021) coupled to a TIMSTOF-FleX (Bruker Daltonics, Bremen, Germany, 2022). Peptides from each sample were diluted using LC–MS Water 0.1% (v/v) formic acid to 10 ng/μL as the final concentration in order to load a total amount of 200 ng of each sample or QC in the Evotips. As indicated above, a pool of samples was created by mixing the same volume of each sample for QC purposes. Pierce Hela Tryptic Digest Standard (Thermo Scientific) was also prepared to ensure system equilibration. The system was calibrated both in mass and in ion mobility dimensions by using a spiked filter with three reference calibrants (lock masses). The reference calibrants are hexakis(1*H*,1*H*,3*H*-tetrafluoropropoxy)phosphazine, hexakis(1*H*,1*H*,5*H*-octafluoropentoxy)phosphazine and hexakis(1*H*,1*H*,7*H*-dodecafluoroheptoxy)phosphazine with corresponding m/z, 1/K0:622.0289 Th, 0.9848 Vs cm^–2^; 922.0097 Th, 1.1895 Vs cm^–2^; 1221.9906 Th, 1.3820 Vs cm^–2^ (Agilent Technologies). The acquisition method used was DIA (Data Independent Analysis) acquisition in the PASEF and PaSER modes.

All MS analyses of samples were randomly performed (to prevent batch effect). For DIA-PASEF acquisitions, the EvosepOne 60SPD chromatography method was used, the mass range was defined from 400.0 to 1201.0 *m*/*z*, and the ion mobility range was set up in two mobility ranges from 0.8 1/K0 to 1.3 1/K0. The collision energy was decreased linearly from 59 eV at 1/K0 = 1.60 Vs cm^–2^ to 20 eV at 1/K0 = Vs cm^–2^. Cycle time was estimated by the system in 1.80 s.

### Data Analysis of QC

Pooled sample analyses acquired in the DIA-PASEF mode were used to monitor system performance during the acquisition of the entire experiment. Puantification of these pooled samples was done using Spectronaut software. The lowest correlation between quantifications observed was 0.9935, and the highest correlation was 0.9963, with an average value of 0.9949, demonstrating that the experimental analysis had high stability during the entire acquisition. To evaluate the reproducibility of the analysis, some random samples were analyzed in duplicate to estimate the experiment’s reproducibility by calculating the correlation for each sample replicate analysis and the first one analyzed. For this purpose, the total intensity area of the first replicate of each sample was plotted on the *X*-axis, and the total intensity area of the replicates of each sample analyzed during the entire experiment was plotted on the *Y*-axis. The reproducibility of the analysis was determined by the *R*^2^ value of each series.

The analysis of chromatography data for the internal QC samples (Figures S1 and S2) demonstrates good reproducibility between runs and confirms the stability of the chromatography across sample acquisitions. To obtain additional QC indicators, the MSDAP R package v1.0.5 (https://github.com/ftwkoopmans/msdap/) was used. This package allowed us to investigate the reproducibility and global clustering of samples by visualizing several QC metrics, including the number of peptides/proteins detected in each sample, data set completeness, local effects in HPLC peptide retention time (RT) per sample, or reproducibility of peptide quantification among replicates (Figures S1 and S2).

### Bioinformatics

Different software was used for network and system biology analysis of the results, which included Ingenuity Pathway Analysis (IPA, Qiagen), Reactome (https://reactome.org), Metascape (https://metascape.org), and String (https://string-db.org). IPA analysis also generates a *z*-score based on the literature to predict activation or inhibition. Fisher’s Exact Test *p*-values ≤0.05 Values greater than or equal to 2 indicate activation, while values less than or equal to −2 indicate inhibition.^14^ The stronger the color, the stronger the *z*-score gray, and no activity pattern is detected.

## Results

### Proteomic Analysis

**Figure 1:**
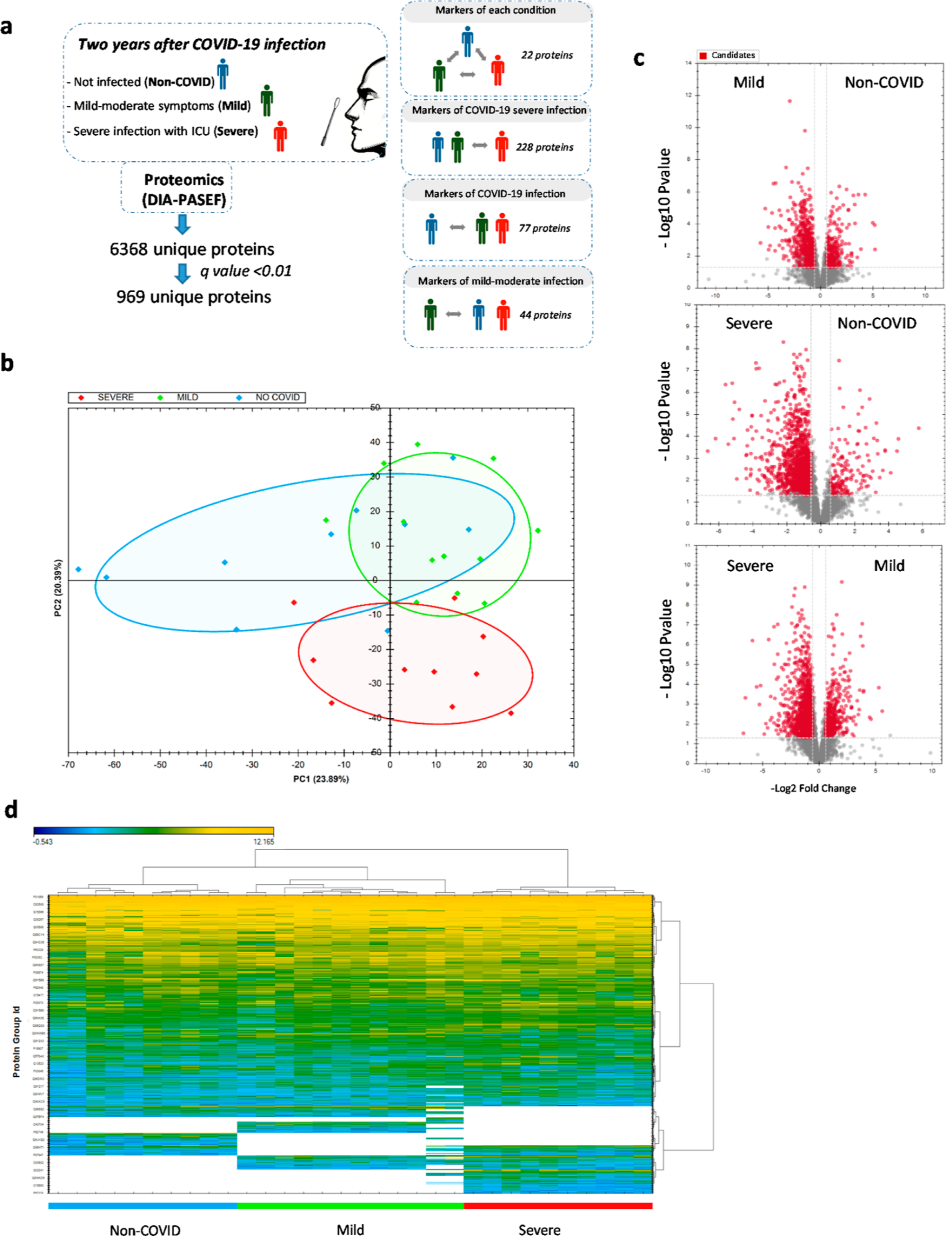
Summary of experiments and proteomic conditions. (a) graphical summary. (b) PCA of the data set of the three experimental conditions. (c) Volcano plots of the comparisons between the three conditions. For biomarker discovery, only proteins are identified in all groups (threshold, *q* value < 0.05). (d) Heatmap.

The whole biomarker discovery study is summarized in Figure 1a. DIA-PASEF proteomics retrieved a total of 6368 unique proteins that was reduced to 2293 when a q < 0.05 was considered. The complete data set with the DIA-PASEF results as well as comparisons between unfiltered protein intensities among non-COVID mild and severe groups can be found in Table S1. Information regarding the internal QC of the whole proteomic study is shown in Figures S1 and S2. PCA successfully reduced the dimensionality of the data while preserving a high proportion of the original variance (Figure 1b). Volcano plot analysis and heatmap of the three experimental conditions showed a higher number of up- or down-regulated proteins in the severe group when compared to non-COVID and Mild groups (Figure 1c,d). In order to reduce the number of false negative results, we focused our attention on those proteins that display a q < 0.01, and only proteins detected in all experimental groups and therefore with a *q* value were considered. Notwithstanding this, a list of proteins not detected in the non-COVID group and those absent in the severe group can be found in Table S1 for informative purposes.

Under the described parameters, we identified a total of 1360 proteins with significant differences between different groups that constitute a total of 969 unique proteins. From all the potential comparisons, we focused our attention on three of them, which we considered the more important for clinical purpose: (1) proteins that were different between all groups and, therefore, would constitute the most valuable biomarkers; (2) proteins that constitute biomarkers of COVID-19 infection that cause severe effects; and (3) proteins that constitute hallmarks of infection with COVID-19, irrespectively of the severity, and therefore markers of an absence of COVID-19 infection (summarized in Figure 1a). Results of IPA analysis for canonical pathways of all of the comparisons are shown in Table S2.

### Proteins Differentially Expressed between Uninfected Patients, Patients with Mild Symptoms, and Those with Severe Infections

**Figure 2:**
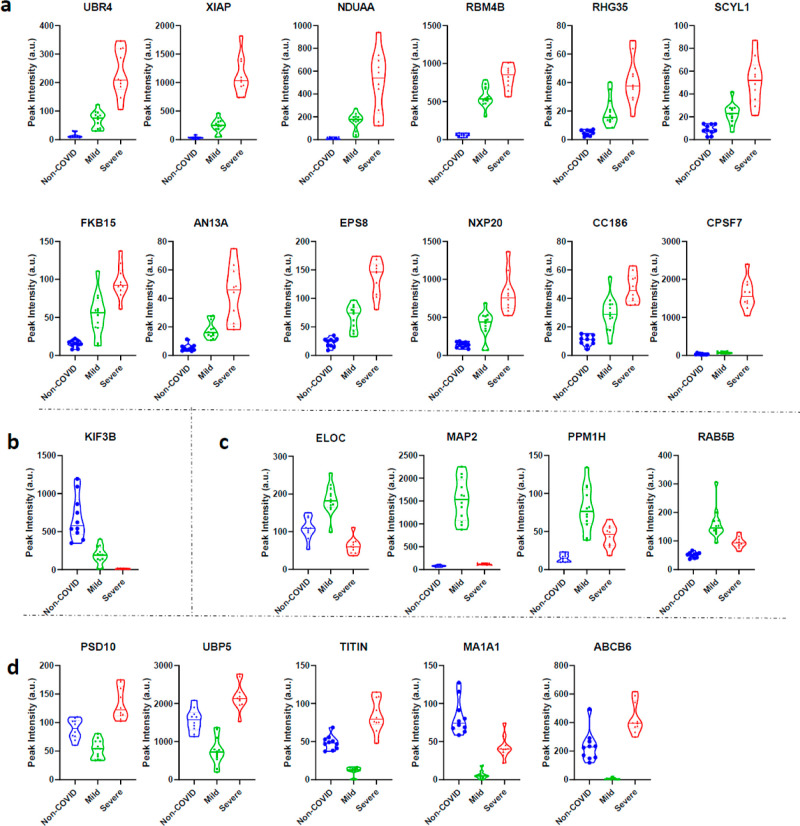
Proteins that were significantly different between the three experimental conditions (severe, mild, and non-COVID samples). Violin plots of the intensity of the different proteins (peptides) within the different conditions. Horizontal black and dash lines indicate the median and interquartile range of the protein abundance. (a) Proteins that appear increased with the severity of the infection. (b) Unique protein that was found to decrease expression with the severity of COVID-19. Proteins that increase (c) or decrease (d) in the mild group.

A set of 22 proteins demonstrated a robust statistic regarding differential expression among the three groups (Table 2). Most of them corresponded to proteins that increased with the severity of the infection (UBR4, XIAP, NDUAA, RBM4B, RHG35, SCYL1, FKB15, AN13A, EPS8, NXP20, CC186, and CPSF7; Figure 2a), and exclusively one protein (KIF3B) displayed opposite behavior (Figure 2b). Another set of proteins was found to change only under the Mild condition. Specifically, 4 proteins increased (elongin C (ELOC), MAP2, PPM1H, and RAB5A; Figure 2c) and 5 proteins decreased (PSD10, UBP5, TITIN, MA1A1, and ABCB6; Figure 2d) in this condition. Bioinformatics analysis only identified one consistent canonical pathway, the ubiquitination pathway, with 5 proteins contributing (ELOC, PSMD10, UBP5 UBR4, and XIAP [IPA, -log(*P* value) = 4 and Reactome analysis). Within this group, it is especially relevant the XIAP protein as it exhibited a 7-fold (q < 0.0019) and 44-fold increase (q < 0.0001) in mild and severe vs non-COVID, respectively.

**Table 2:** Description of the 22 Proteins That Were Found Differentially Expressed between all Group Proteins (*q* < 0.01)

protein (human)	protein description	fold change in non-COVID vs mild	fold change in non-COVID vs severe	fold change in mild vs severe	genes	UniProt Ids
MAP2	methionine aminopeptidase 2	–19.3	–1.5	12.8	METAP2	P50579
NDUAA	NADH dehydrogenase [ubiquinone] 1 alpha subcomplex subunit 10, mitochondrial	–13.6	–43.4	–3.2	NDUFA10	O95299
RBM4B	RNA-binding protein 4B	–9.6	–14.1	–1.5	RBM4B	Q9BQ04
XIAP	E3 ubiquitin-protein ligase XIAP	–7.5	–33.8	–4.5	XIAP	P98170
PPM1H	protein phosphatase 1H	–5.1	–2.8	1.8	PPM1H	Q9ULR3
UBR4	E3 ubiquitin-protein ligase UBR4	–5.0	–16.5	–3.3	UBR4	Q5T4S7
RHG35	Rho GTPase-activating protein 35	–4.0	–8.5	–2.1	ARHGAP35	Q9NRY4
FKB15	FK506-binding protein 15	–3.5	–6.4	–1.8	FKBP15	Q5T1M5
AN13A	ankyrin repeat domain-containing protein 13A	–3.4	–8.1	–2.4	ANKRD13A	Q8IZ07
RAB5B	ras-related protein Rab-5B	–3.1	–1.9	1.7	RAB5B	P61020
EPS8	epidermal growth factor receptor kinase substrate 8	–3.0	–6.0	–2.0	EPS8	Q12929
NXP20	protein NOXP20	–2.9	–5.8	–2.0	FAM114A1	Q8IWE2
CC186	coiled-coil domain-containing protein 186	–2.8	–4.4	–1.6	CCDC186	Q7Z3E2
SCYL1	N-terminal kinase-like protein	–2.6	–5.8	–2.3	SCYL1	Q96KG9
CPSF7	cleavage and polyadenylation specificity factor subunit 7	–2.2	–48.5	–21.6	CPSF7	Q8N684
ELOC	elongin-C	–1.7	1.8	3.0	ELOC	Q15369
PSD10	26S proteasome non-ATPase regulatory subunit 10	1.7	–1.5	–2.5	PSMD10	O75832
UBP5	ubiquitin carboxyl-terminal hydrolase 5	2.1	–1.4	–2.9	USP5	P45974
TITIN	titin	3.5	–1.7	–6.1	TTN	Q8WZ42
KIF3B	kinesin-like protein KIF3B	3.6	54.6	15.2	KIF3B	O15066
MA1A1	mannosyl-oligosaccharide 1,2-alpha-mannosidase IA	12.4	1.9	–6.5	MAN1A1	P33908
ABCB6	ATP-binding cassette subfamily B member 6	31.8	–1.8	–58.7	ABCB6	Q9NP58

### Protein Biomarkers of SARS-CoV-2 Infection Associated with Severe Effects

**Figure 3:**
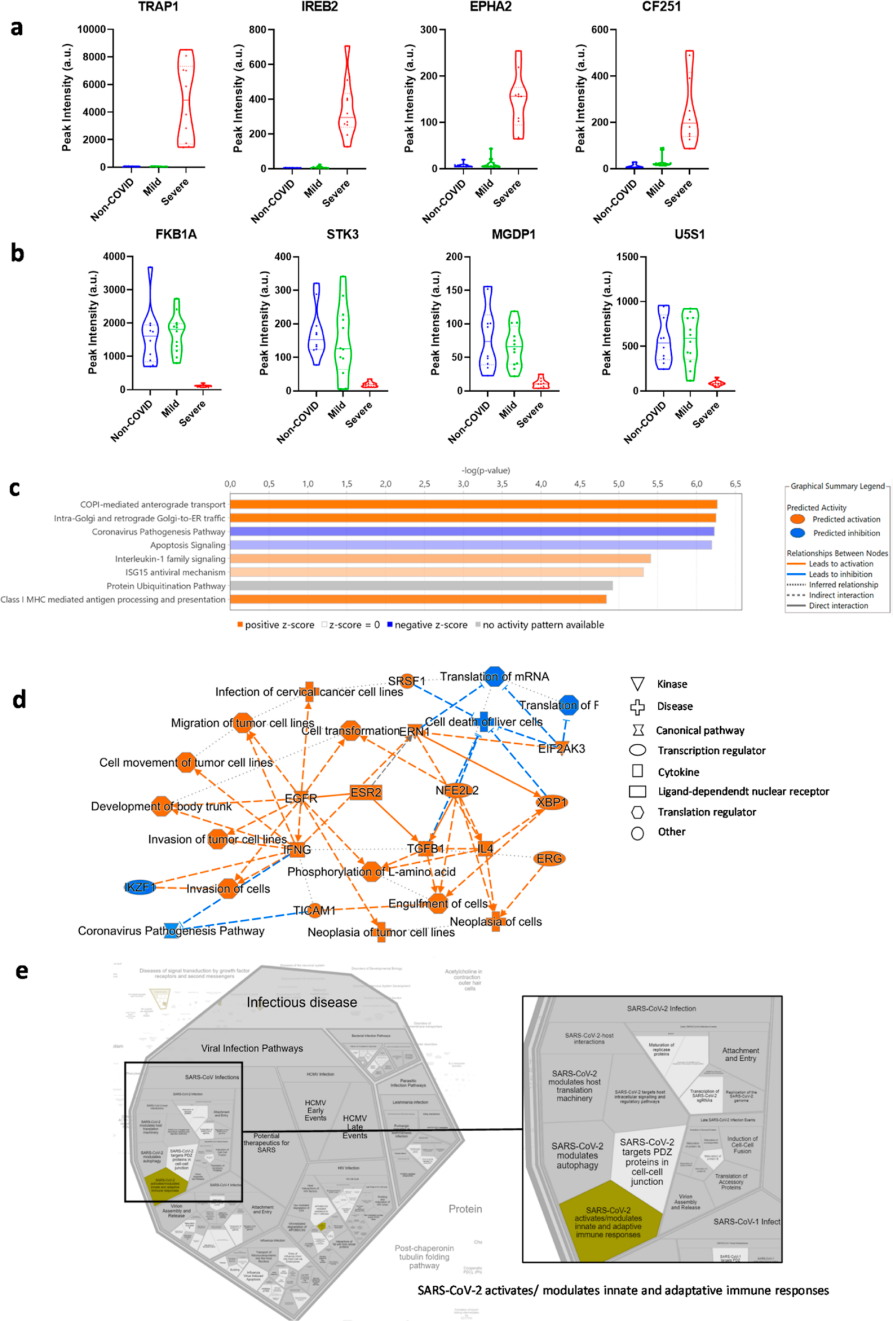
Biomarkers of SARS-CoV-2 severe infections. (a) Representative violin plots of 4 out of 280 proteins that were found overexpressed in severe conditions. (b) Representative violin plots of 4 out of 26 proteins that were down-regulated in the severe group. (c,d) Bioinformatics of the results allowed for the identification of several canonical pathways that were predicted to be increased (orange; positive *z*-score) or repressed (blue; negative *z*-score), −log (*P* value) > 4.5. Fisheŕs exact test *p*-value. (e) When infectious diseases were investigated using Reactome, SARS-CoV-2 modulation of the immune response was identified. Statistical data can be found in Table S2.

We aimed at identifying those proteins that constitute hallmarks of patients who suffered from severe COVID-19 infections in order to differentiate them from mild and non-COVID patients. Interestingly, this study was the one that retrieved more proteins, and a total of 228 proteins were found differentially expressed, being mostly up-regulated in severe COVID (202) but also down-regulated (26 proteins) vs both mild and non-COVID samples (Table 3). The distribution of the peak intensities for 4 representative proteins from each group is represented in Figure 3a,b. Bioinformatics analysis of these results considering up- or down-regulation of the proteins in patients admitted to the ICU using IPA identified several canonical pathways with a positive *z*-score (Figure 3c), mostly related anterograde, retrograde, and intra-Golgi transport. Among them, we can highlight proteins of the conserved oligomeric Golgi (COG) complex (COG2, COG3, and COG7) that, along with RAB6A and proteins of the dynein/dynactin motor complex DCTN1 and DCTN4, control retrograde transport.^15^

**Table 3:** Description of the 228 Proteins That Were Found Differentially Expressed in Severe COVID-19 Infections vs the Other Groups (*q* < 0.01)^a^

protein (human)	protein description	fold change in severe vs mild	fold change in severe vs non-COVID	genes	Uniprot Ids
FKB1A	peptidyl-prolyl cis–trans isomerase FKBP1A	–14.67	–13.85	FKBP1A	P62942
STK3	serine/threonine-protein kinase 3	–7.44	–8.79	STK3	Q13188
MGDP1	magnesium-dependent phosphatase 1	–5.97	–7.16	MDP1	Q86 V88
CFDP1	craniofacial development protein 1	–5.17	–6.76	CFDP1	Q9UEE9
U5S1	116 kDa U5 small nuclear ribonucleoprotein component	–6.44	–6.46	EFTUD2	Q15029
ST2B1	sulfotransferase 2B1	–6.40	–6.24	SULT2B1	O00204
CCD91	coiled-coil domain-containing protein 91	–4.86	–5.59	CCDC91	Q7Z6B0
CNOT3	CCR4-NOT transcription complex subunit 3	–4.73	–4.79	CNOT3	O75175
IMDH1#	inosine-5′-monophosphate dehydrogenase 1	–5.77	–4.66	IMPDH1	P20839
TCEA1	transcription elongation factor A protein 1	–5.20	–4.56	TCEA1	P23193
CRBG1	β/γ crystallin domain-containing protein 1	–4.28	–4.42	CRYBG1	Q9Y4K1
SKT#	sickle tail protein homologue	–2.17	–3.92	KIAA1217	Q5T5P2
PCDH1	protocadherin-1	–2.53	–3.24	PCDH1	Q08174
SRSF6	serine/arginine-rich splicing factor 6	–4.37	–3.06	SRSF6	Q13247
BAX*	apoptosis regulator BAX	–2.06	–2.92	BAX	Q07812
VPS25	vacuolar protein-sorting-associated protein 25	–2.38	–2.73	VPS25	Q9BRG1
HDHD1	pseudouridine-5′-phosphatase	–3.27	–2.72	PUDP	Q08623
NUP37	nucleoporin Nup37	–3.01	–2.45	NUP37	Q8NFH4
UB2 V1	ubiquitin-conjugating enzyme E2 variant 1	–2.58	–2.41	UBE2 V1	Q13404
CUX1	homeobox protein cut-like 1	–3.52	–2.38	CUX1	P39880
MTNA	methylthioribose-1-phosphate isomerase	–2.28	–2.18	MRI1	Q9BV20
SNX1#	sorting nexin-1	–1.99	–2.10	SNX1	Q13596
EZRI	ezrin	–2.38	–2.06	EZR	P15311
UBE2N	ubiquitin-conjugating enzyme E2 N	–2.20	–2.03	UBE2N	P61088
H1BP3	HCLS1-binding protein 3	–1.64	–1.54	HS1BP3	Q53T59
RPB3	DNA-directed RNA polymerase II subunit RPB3	–1.50	–1.49	POLR2C	P19387
SYSC	serine-tRNA ligase, cytoplasmic	1.54	1.29	SARS1	P49591
MK03*	mitogen-activated protein kinase 3	1.53	1.32	MAPK3	P27361
GUAA	GMP synthase [glutamine-hydrolyzing]	2.10	1.36	GMPS	P49915
HSP7E	heat shock 70 kDa protein 14	1.82	1.40	HSPA14	Q0VDF9
CSK21	casein kinase II subunit alpha	1.37	1.42	CSNK2A1	P68400
CSDE1	cold shock domain-containing protein E1	1.61	1.42	CSDE1	O75534
FUBP2	far upstream element-binding protein 2	2.44	1.43	KHSRP	Q92945
KLC1	kinesin light chain 1	1.66	1.44	KLC1	Q07866
EF1G	elongation factor 1-γ	1.59	1.45	EEF1G	P26641
SLK	STE20-like serine/threonine-protein kinase	1.46	1.45	SLK	Q9H2G2
EIF2A*	eukaryotic translation initiation factor 2A	2.05	1.46	EIF2A	Q9BY44
CTBP1	C-terminal-binding protein 1	1.93	1.48	CTBP1	Q13363
SYCC	cysteine-tRNA ligase, cytoplasmic	2.06	1.49	CARS1	P49589
GRAP1	GRIP1-associated protein 1	1.53	1.49	GRIPAP1	Q4 V328
PTN11	tyrosine-protein phosphatase nonreceptor type 11	2.21	1.50	PTPN11	Q06124
DCTN1#	dynactin subunit 1	1.92	1.51	DCTN1	Q14203
FAAA	fumarylacetoacetase	1.38	1.52	FAH	P16930
IMB1*	importin subunit β-1	1.78	1.55	KPNB1	Q14974
PP2BA	protein phosphatase 3 catalytic subunit α	1.37	1.57	PPP3CA	Q08209
PSA	puromycin-sensitive aminopeptidase	1.71	1.59	NPEPPS	P55786
PSMD8	26S proteasome non-ATPase regulatory subunit 8	1.30	1.60	PSMD8	P48556
COG7#	conserved oligomeric Golgi complex subunit 7	1.85	1.61	COG7	P83436
CSN4#	COP9 signalosome complex subunit 4	1.92	1.62	COPS4	Q9BT78
ERF3A	eukaryotic peptide chain release factor GTP-binding subunit ERF3A	1.82	1.63	GSPT1	P15170
SETD7	histone-lysine N-methyltransferase SETD7	1.89	1.64	SETD7	Q8WTS6
ANS1A	ankyrin repeat and SAM domain-containing protein 1A	1.90	1.65	ANKS1A	Q92625
AIMP2	aminoacyl tRNA synthase complex-interacting multifunctional protein 2	1.65	1.65	AIMP2	Q13155
RBM47	RNA-binding protein 47	1.60	1.65	RBM47	A0AV96
HNRH1	heterogeneous nuclear ribonucleoprotein H	1.35	1.68	HNRNPH1	P31943
DX39A	ATP-dependent RNA helicase DDX39A	1.40	1.69	DDX39A	O00148
SEPT8	septin-8	1.92	1.71	SEPTIN8	Q92599
LPP#	lipoma-preferred partner	1.70	1.72	LPP	Q93052
KINH	kinesin-1 heavy chain	1.69	1.73	KIF5B	P33176
GAK	cyclin-G-associated kinase	1.54	1.73	GAK	O14976
SAHH3#	adenosylhomocysteinase 3	1.79	1.75	AHCYL2	Q96HN2
IPO5	importin-5	3.98	1.76	IPO5	O00410
MCTS1#	malignant T-cell-amplified sequence 1	1.74	1.76	MCTS1	Q9ULC4
NADAP#	kanadaptin	1.50	1.76	SLC4A1AP	Q9BWU0
PYRG2	CTP synthase 2	2.19	1.76	CTPS2	Q9NRF8
MCES	mRNA cap guanine-N7 methyltransferase	1.67	1.79	RNMT	O43148
USO1	general vesicular transport factor p115	3.01	1.79	USO1	O60763
BUB3	mitotic checkpoint protein BUB3	1.55	1.83	BUB3	O43684
IF2G	eukaryotic translation initiation factor 2 subunit 3	1.66	1.83	EIF2S3	P41091
ACTN1	α-actinin-1	1.94	1.84	ACTN1	P12814
KDM3B	lysine-specific demethylase 3B	1.49	1.85	KDM3B	Q7LBC6
TBCE	tubulin-specific chaperone E	1.55	1.87	TBCE	Q15813
SYNC	asparagine-tRNA ligase, cytoplasmic	2.16	1.87	NARS1	O43776
PLST	plastin-3	2.19	1.88	PLS3	P13797
DDX17	probable ATP-dependent RNA helicase DDX17	1.82	1.89	DDX17	Q92841
COG2	conserved oligomeric Golgi complex subunit 2	1.49	1.90	COG2	Q14746
UEVLD	ubiquitin-conjugating enzyme E2 variant 3	1.94	1.91	UEVLD	Q8IX04
ES8L2	epidermal growth factor receptor kinase substrate 8-like protein 2	2.39	1.92	EPS8L2	Q9H6S3
PUS1	pseudouridylate synthase 1 homologue	1.44	1.93	PUS1	Q9Y606
PPR21	protein phosphatase 1 regulatory subunit 21	1.69	1.95	PPP1R21	Q6ZMI0
RS2*	40S ribosomal protein S2	1.82	1.97	RPS2	P15880
XPO2	exportin-2	1.91	1.98	CSE1L	P55060
STAM1	signal transd1 ng adapter molecule 1	1.70	1.98	STAM	Q92783
PTN6	tyrosine-protein phosphatase nonreceptor type 6	2.03	1.98	PTPN6	P29350
RAGP1	Ran GTPase-activating protein 1	1.57	1.99	RANGAP1	P46060
LRBA#	lipopolysaccharide-responsive and beige-like anchor protein	2.49	2.00	LRBA	P50851
ECM29	proteasome adapter and scaffold protein ECM29	1.95	2.00	ECPAS	Q5VYK3
SAHH2	*S*-adenosylhomocysteine hydrolase-like protein 1	1.82	2.02	AHCYL1	O43865
NFKB2*	nuclear factor NF-kappa-B p100 subunit	2.07	2.02	NFKB2	Q00653
EIF3H	eukaryotic translation initiation factor 3 subunit H	3.23	2.03	EIF3H	O15372
IF16	γ-interferon-ind1ble protein 16	1.84	2.03	IFI16	Q16666
DCAF1	DDB1- and CUL4-associated factor 1	1.81	2.06	DCAF1	Q9Y4B6
ABCE1	ATP-binding cassette subfamily E member 1	1.92	2.06	ABCE1	P61221
KCC2D	calcium/calmodulin-dependent protein kinase type II subunit delta	2.03	2.08	CAMK2D	Q13557
FBX22	F-box only protein 22	2.46	2.09	FBXO22	Q8NEZ5
CASP7	caspase-7	2.28	2.09	CASP7	P55210
SF3A3	splicing factor 3A subunit 3	1.67	2.10	SF3A3	Q12874
DLG1	disks large homologue 1	1.40	2.10	DLG1	Q12959
LAMP2	lysosome-associated membrane glycoprotein 2	2.06	2.10	LAMP2	P13473
ACAP2	Arf-GAP with coiled-coil, ANK repeat and PH domain-containing protein 2	2.25	2.10	ACAP2	Q15057
AMPN	aminopeptidase N	2.35	2.11	ANPEP	P15144
VP26B	vacuolar protein sorting-associated protein 26B	2.71	2.11	VPS26B	Q4G0F5
SC24D	protein transport protein Sec24D	2.40	2.11	SEC24D	O94855
SND1	staphylococcal nuclease domain-containing protein 1	3.76	2.12	SND1	Q7KZF4
NEUL	neurolysin, mitochondrial	3.53	2.12	NLN	Q9BYT8
CBX1	chromobox protein homologue 1	2.04	2.13	CBX1	P83916
MAP1S	microtubule-associated protein 1S	1.97	2.13	MAP1S	Q66K74
MTURN	maturin	2.55	2.14	MTURN	Q8N3F0
VP26C	vacuolar protein sorting-associated protein 26C	3.04	2.14	VPS26C	O14972
PSMD1	26S proteasome non-ATPase regulatory subunit 1	2.96	2.17	PSMD1	Q99460
RANB9	ran-binding protein 9	2.61	2.17	RANBP9	Q96S59
DHX36	ATP-dependent DNA/RNA helicase DHX36	2.96	2.22	DHX36	Q9H2U1
COG3#	conserved oligomeric Golgi complex subunit 3	2.10	2.24	COG3	Q96JB2
ACSA	acetyl-coenzyme A synthetase, cytoplasmic	1.98	2.24	ACSS2	Q9NR19
DEOC	deoxyribose-phosphate aldolase	2.00	2.24	DERA	Q9Y315
RN5A	2–5A-dependent ribonuclease	2.19	2.26	RNASEL	Q05823
GTPB1	GTP-binding protein 1	3.47	2.26	GTPBP1	O00178
AP1B1	AP-1 complex subunit β-1	2.72	2.28	AP1B1	Q10567
FCHO2	F-BAR domain only protein 2	2.24	2.28	FCHO2	Q0JRZ9
EXOC5	exocyst complex component 5	1.93	2.29	EXOC5	O00471
FLNB	filamin-B	2.55	2.30	FLNB	O75369
MB12A	multivesicular body subunit 12A	1.91	2.30	MVB12A	Q96EY5
DDX3X	ATP-dependent RNA helicase DDX3X	1.95	2.31	DDX3X	O00571
RENT1	regulator of nonsense transcripts 1	2.78	2.34	UPF1	Q92900
SEPT11	septin-11	1.47	2.34	SEPTIN11	Q9NVA2
PARP4	protein mono-ADP-ribosyltransferase PARP4	2.44	2.35	PARP4	Q9UKK3
RAB1B	ras-related protein Rab-1B	2.28	2.36	RAB1B	Q9H0U4
SH24A	SH2 domain-containing protein 4A	1.85	2.36	SH2D4A	Q9H788
EI2BA	translation initiation factor eIF-2B subunit alpha	2.25	2.37	EIF2B1	Q14232
SRXN1	sulfiredoxin-1	2.21	2.45	SRXN1	Q9BYN0
UBP8	ubiquitin carboxyl-terminal hydrolase 8	2.94	2.46	USP8	P40818
SIR2	NAD-dependent protein deacetylase sirtuin-2	3.07	2.49	SIRT2	Q8IXJ6
ZCCHV*	zinc finger CCCH-type antiviral protein 1	1.97	2.49	ZC3HAV1	Q7Z2W4
NS1BP	influenza virus NS1A-binding protein	3.09	2.51	IVNS1ABP	Q9Y6Y0
INO1	inositol-3-phosphate synthase 1	1.76	2.51	ISYNA1	Q9NPH2
JIP4#	C-Jun-amino-terminal kinase-interacting protein 4	3.61	2.52	SPAG9	O60271
TBK1*	serine/threonine-protein kinase TBK1	2.65	2.52	TBK1	Q9UHD2
UBE3C#	ubiquitin-protein ligase E3C	4.79	2.55	UBE3C	Q15386
DCTN4#	dynactin subunit 4	2.24	2.55	DCTN4	Q9UJW0
UBP24	ubiquitin carboxyl-terminal hydrolase 24	2.41	2.56	USP24	Q9UPU5
PYRG1	CTP synthase 1	3.26	2.57	CTPS1	P17812
MY18A	unconventional myosin-XVIIIa	2.89	2.57	MYO18A	Q92614
MRP1	multidrug resistance-associated protein 1	7.87	2.59	ABCC1	P33527
DPP3	dipeptidyl peptidase 3	2.56	2.59	DPP3	Q9NY33
G6PD	glucose-6-phosphate 1-dehydrogenase	2.60	2.60	G6PD	P11413
RIGI*	antiviral innate immune response receptor RIG-I	2.68	2.63	RIGI	O95786
OSBL2	oxysterol-binding protein-related protein 2	2.07	2.67	OSBPL2	Q9H1P3
PACS1	phosphofurin acidic cluster sorting protein 1	1.62	2.68	PACS1	Q6VY07
SC23A	protein transport protein Sec23A	1.74	2.73	SEC23A	Q15436
OPLA#	5-oxoprolinase	2.30	2.77	OPLAH	O14841
ABR	active breakpoint cluster region-related protein	3.07	2.77	ABR	Q12979
SYIC	isole1ne-tRNA ligase, cytoplasmic	1.98	2.77	IARS1	P41252
KI13B	kinesin-like protein KIF13B	2.02	2.82	KIF13B	Q9NQT8
ARHG7	Rho guanine nucleotide exchange factor 7	1.92	2.82	ARHGEF7	Q14155
GOGA3	Golgi subfamily A member 3	1.69	2.85	GOLGA3	Q08378
HXK2	hexokinase-2	3.20	2.87	HK2	P52789
AP3M1	AP-3 complex subunit mu-1	5.88	2.87	AP3M1	Q9Y2T2
DAG1	dystroglycan 1	2.00	2.88	DAG1	Q14118
UGGG1	UDP-glucose:glycoprotein glucosyltransferase 1	2.80	2.88	UGGT1	Q9NYU2
GPD1L	glycerol-3-phosphate dehydrogenase 1-like protein	3.59	2.88	GPD1L	Q8N335
RS8*#	40S ribosomal protein S8	1.84	2.89	RPS8	P62241
RAB6A	ras-related protein Rab-6A	1.71	2.91	RAB6A	P20340
DCA11	DDB1- and CUL4-associated factor 11	2.60	2.92	DCAF11	Q8TEB1
RELCH	RAB11-binding protein RELCH	4.79	2.94	RELCH	Q9P260
PTN23	tyrosine-protein phosphatase nonreceptor type 23	4.30	2.94	PTPN23	Q9H3S7
ARL2	ADP-ribosylation factor-like protein 2	1.85	2.99	ARL2	P36404
INF2	inverted formin-2	3.23	2.99	INF2	Q27J81
MYO1D	unconventional myosin-Id	2.89	3.04	MYO1D	O94832
RAP2B	ras-related protein Rap-2b	2.26	3.05	RAP2B	P61225
CUL4B	cullin-4B	1.69	3.05	CUL4B	Q13620
IF2P	eukaryotic translation initiation factor 5B	1.82	3.06	EIF5B	O60841
NU133	nuclear pore complex protein Nup133	2.23	3.11	NUP133	Q8WUM0
REL*	proto-oncogene c-Rel	2.67	3.18	REL	Q04864
CDK5	cyclin-dependent kinase 5	3.13	3.23	CDK5	Q00535
DJC10	DnaJ homologue subfamily C member 10	3.83	3.24	DNAJC10	Q8IXB1
NEUA	*N*-acylneuraminate cytidylyltransferase	3.07	3.25	CMAS	Q8NFW8
PROM1	prominin-1	2.94	3.26	PROM1	O43490
CF132	uncharacterized protein C6orf132	2.04	3.29	C6orf132	Q5T0Z8
CUL2	cullin-2	2.98	3.32	CUL2	Q13617
VPS50	syndetin	4.98	3.38	VPS50	Q96JG6
STXB2	syntaxin-binding protein 2	2.30	3.41	STXBP2	Q15833
VPS39	Vam6/Vps39-like protein	2.90	3.41	VPS39	Q96JC1
ACSL1	long-chain-fatty-acid-CoA ligase 1	3.19	3.42	ACSL1	P33121
SPTN1	spectrin alpha chain, nonerythrocytic 1	2.96	3.46	SPTAN1	Q13813
PRP6	Pre-mRNA-processing factor 6	3.90	3.48	PRPF6	O94906
SRPRA	signal recognition particle receptor subunit α	3.18	3.52	SRPRA	P08240
PTK6	protein-tyrosine kinase 6	3.59	3.52	PTK6	Q13882
NXN	nucleoredoxin	7.62	3.60	NXN	Q6DKJ4
CAN5	calpain-5	3.73	3.71	CAPN5	O15484
SPTN2	spectrin β chain, nonerythrocytic 2	8.84	3.78	SPTBN2	O15020
TBC23	TBC1 domain family member 23	4.17	3.82	TBC1D23	Q9NUY8
FBLN1	fibulin-1	2.61	3.82	FBLN1	P23142
DEN10	DENN domain-containing protein 10	2.67	3.85	DENND10	Q8TCE6
MA2A1#	α-mannosidase 2	6.00	3.93	MAN2A1	Q16706
MANBA	β-mannosidase	6.27	4.06	MANBA	O00462
ABCF3	ATP-binding cassette subfamily F member 3	2.49	4.15	ABCF3	Q9NUQ8
DAA10	dynein axonemal assembly factor 10	4.89	4.20	DNAAF10	Q96MX6
SCRB2	lysosome membrane protein 2	2.24	4.22	SCARB2	Q14108
AKAP8	A-kinase anchor protein 8	15.03	4.39	AKAP8	O43823
PPAP	prostatic acid phosphatase	7.75	4.41	ACP3	P15309
FXL18#	F-box/LRR-repeat protein 18	4.58	4.58	FBXL18	Q96ME1
ANXA9	annexin A9	5.51	4.77	ANXA9	O76027
STRBP	spermatid perinuclear RNA-binding protein	2.52	4.79	STRBP	Q96SI9
EMAL3	echinoderm microtubule-associated protein-like 3	2.44	5.21	EML3	Q32P44
VAV	proto-oncogene vav	3.00	5.31	VAV1	P15498
RRP44	exosome complex exonuclease RRP44	5.29	5.36	DIS3	Q9Y2L1
SAMD9	sterile alpha motif domain-containing protein 9	4.25	5.63	SAMD9	Q5K651
THOC1	THO complex subunit 1	7.95	6.12	THOC1	Q96FV9
SRP68	signal recognition particle subunit SRP68	2.57	6.24	SRP68	Q9UHB9
IKKA*	inhibitor of nuclear factor kappa-B kinase subunit α	6.27	6.66	CHUK	O15111
ARRB2	β-arrestin-2	10.08	7.09	ARRB2	P32121
AT12A	potassium-transporting ATPase α chain 2	3.31	7.24	ATP12A	P54707
CERT	ceramide transfer protein	4.50	8.52	CERT1	Q9Y5P4
FAF2	FAS-associated factor 2	9.57	8.93	FAF2	Q96CS3
ARMD3	armadillo-like helical domain-containing protein 3	12.52	9.66	ARMH3	Q5T2E6
DOP2	protein dopey-2	6.51	10.90	DOP1B	Q9Y3R5
GPI8	GPI-anchor transamidase	4.90	10.92	PIGK	Q92643
CARD6	caspase recruitment domain-containing protein 6	15.38	11.34	CARD6	Q9BX69
UBP11	ubiquitin carboxyl-terminal hydrolase 11	12.53	12.31	USP11	P51784
EXD2	exonuclease 3′–5′ domain-containing protein 2	2.52	13.82	EXD2	Q9NVH0
ZFR	zinc finger RNA-binding protein	7.34	13.93	ZFR	Q96KR1
DTX3L	E3 ubiquitin-protein ligase DTX3L	9.84	16.54	DTX3L	Q8TDB6
MTA2	metastasis-associatedprotein MTA2	12.11	17.09	MTA2	O94776
EPHA2	ephrin type-A receptor 2	13.37	19.75	EPHA2	P29317
TBCD1	TBC1 domain family member 1	8.39	20.51	TBC1D1	Q86TI0
CF251	cilia- and flagella-associated protein 251	8.38	20.88	CFAP251	Q8TBY9
IREB2	iron-responsive element-binding protein 2	45.92	72.62	IREB2	P48200
TRAP1	heat shock protein 75 kDa, mitochondrial	90.41	100.61	TRAP1	Q12931

a *Proteins identified as part of the coronavirus pathogenesis pathway. # proteins predicted to be regulated by the transcription factor CUX1.

Interestingly, only two pathways appeared with a negative *z*-score and therefore were predicted to be repressed, the most significant being the coronavirus infection pathways (Figure 3c), which was indeed the only one related with infection diseases (Reactome, Figure 3d). All of this information is represented in the IPA graphical overview (Figure 3e). It is interesting to mention that TRAP1 and FKBP1A were the most up-regulated and down-regulated proteins in this study (Table 3) with extremely high fold change (over 90 and −14 times, respectively).

**Figure 4:**
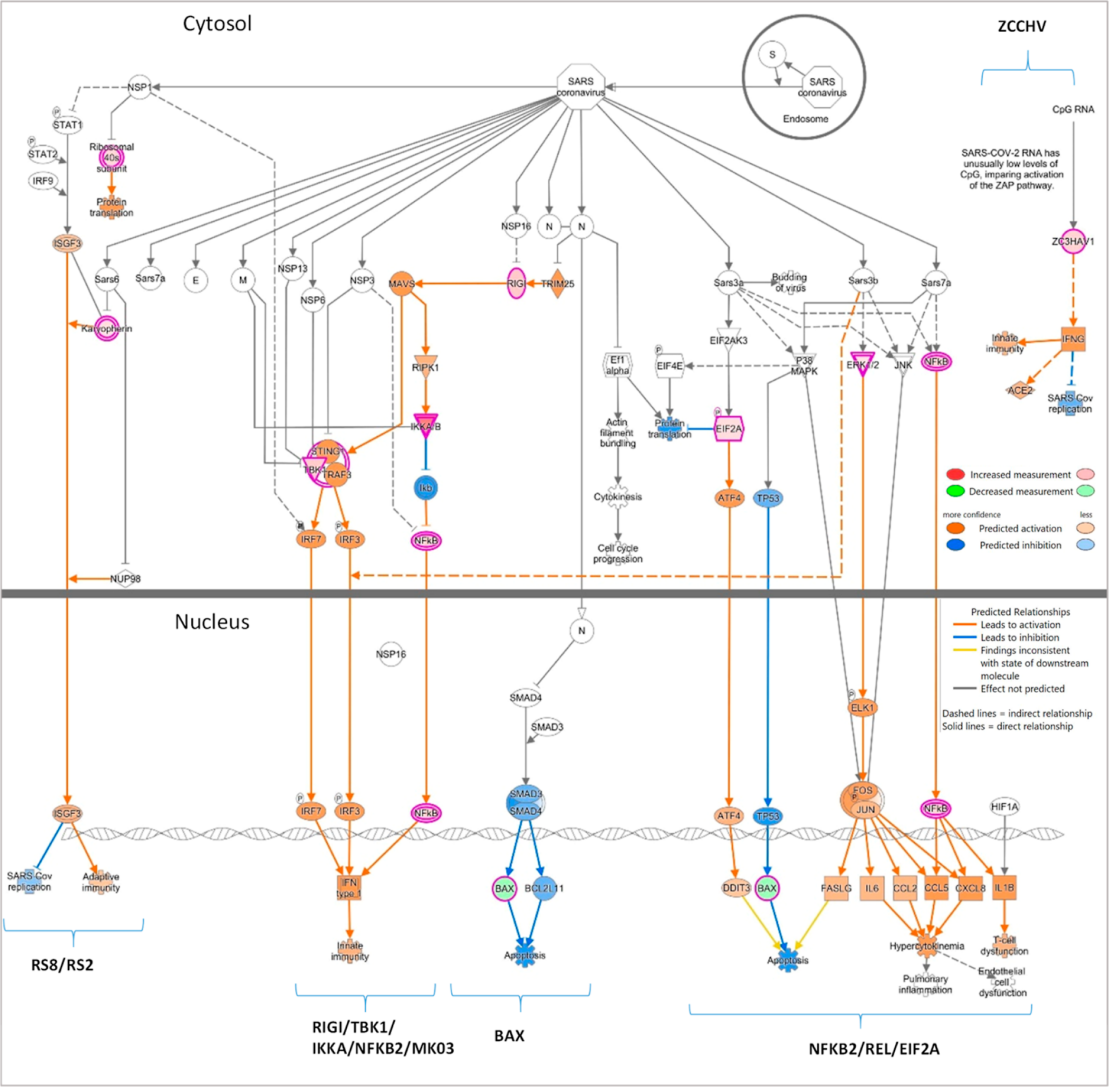
Coronavirus infection pathway (IPA). Proteins up or down-regulated in the severe group and non-COVID that participate in the coronavirus infection pathway. Legends are embedded in the figure. Gene and protein names are included in the pathway.

Interestingly, all the proteins that were included in the coronavirus infection pathway (BAX, IMB1, EIF2A, IKKA, MK03, NFKB2, REL, RIGI, RS2, RS8, TBK1, and ZCCHV; * in Table 3) participate exclusively in intracellular events, as shown in the IPA canonical pathway (Figure 4).

### Proteins that Define the Noninfected Individuals vs Mild and Severe COVID-19 Infections

**Figure 5:**
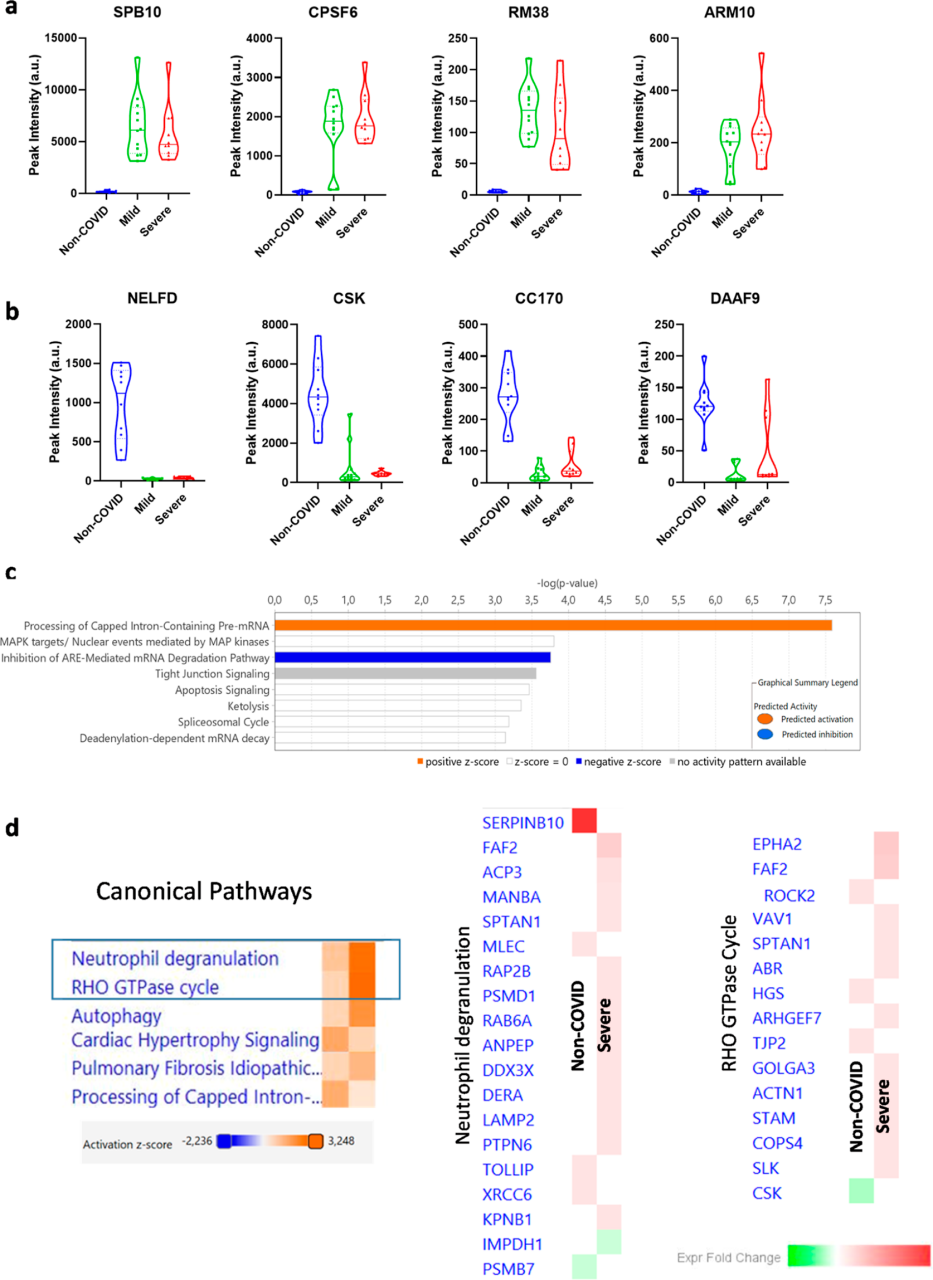
Violin plots of 4 representative proteins found overexpressed (a) or reduced (b) in mild and severe SARS-CoV-2 infections vs the non-COVID group. (c,d) Canonical pathways identified using IPA analysis are shown. Positive *z*-score in orange and negative in blue. Statistical data can be found in Table S2.

We have also examined the data set in search of potential biomarkers of Sars-Cov-2 infection irrespectively of the severity of the process, markers of people that were not infected by Sars-Cov-2. We identified 77 proteins that changed in both mild and severe conditions vs controls (Table 4), wherein 67 were upregulated and 10 were down-regulated in both conditions vs controls. Representative proteins of both up- and down-regulated proteins are shown in Figure 5a,b, respectively. Most of the proteins identified were related to regulatory events of mRNA as depicted from IPA canonical pathway analysis (Figure 5c, Table S2).

**Table 4:** Description of the 77 Proteins That Were Found Differentially Expressed in Severe COVID-19 Infections vs Other Groups (*q* < 0.01)

protein (human)	protein descriptions	fold change in severe vs non-COVID	fold change in moderate vs non-COVID	genes	Uniprot Ids
SPB10	serpin B10	34.53	38.58	SERPINB10	P48595
CPSF6	cleavage and polyadenylation specificity factor subunit 6	27.77	24.52	CPSF6	Q16630
RM38	39S ribosomal protein L38, mitochondrial	17.34	22.32	MRPL38	Q96DV4
FXR1	RNA-binding protein FXR1	16.14	15.20	FXR1	P51114
ARM10	armadillo repeat-containing protein 10	20.02	15.18	ARMC10	Q8N2F6
PKHA2	pleckstrin homology domain-containing family A member 2	4.49	8.59	PLEKHA2	Q9HB19
SCOT1	succinyl-CoA:3-ketoacid coenzyme A transferase 1, mitochondrial	7.48	7.30	OXCT1	P55809
ERLEC	endoplasmic reticulum lectin 1	4.30	6.59	ERLEC1	Q96DZ1
SMHD1	structural maintenance of chromosomes flexible hinge domain-containing protein 1	14.10	6.28	SMCHD1	A6NHR9
GNL1	guanine nucleotide-binding protein-like 1	7.57	4.87	GNL1	P36915
ECHA	trifunctional enzyme subunit alpha, mitochondrial	4.53	4.81	HADHA	P40939
ATRAP	type-1 angiotensin II receptor-associated protein	6.59	4.58	AGTRAP	Q6RW13
MROH1	maestro heat-like repeat-containing protein family member 1	4.38	4.35	MROH1	Q8NDA8
LRC40	Le1ne-rich repeat-containing protein 40	7.47	4.31	LRRC40	Q9H9A6
ROCK2	Rho-associated protein kinase 2	7.27	4.20	ROCK2	O75116
MK08	mitogen-activated protein kinase 8	4.07	4.06	MAPK8	P45983
XRN1	5′–3′ exoribonuclease 1	3.90	3.76	XRN1	Q8IZH2
PKHF2	pleckstrin homology domain-containing family F member 2	3.99	3.72	PLEKHF2	Q9H8W4
SRRT	serrate RNA effector molecule homologue	2.28	3.70	SRRT	Q9BXP5
SF3B4	splicing factor 3B subunit 4	3.41	3.69	SF3B4	Q15427
CPT2	carnitine *O*-palmitoyltransferase 2, mitochondrial	3.06	3.60	CPT2	P23786
FHI2A	FHF complex subunit HOOK interacting protein 2A	5.67	3.41	FHIP2A	Q5W0 V3
GTF2I	general transcription factor II–I	2.14	3.36	GTF2I	P78347
HGS	hepatocyte growth factor-regulated tyrosine kinase substrate	2.23	3.32	HGS	O14964
TARA	TRIO and F-actin-binding protein	3.51	3.23	TRIOBP	Q9H2D6
XPO7	exportin-7	3.74	3.14	XPO7	Q9UIA9
MTMR5	myotubularin-related protein 5	4.08	3.04	SBF1	O95248
PTK7	inactive tyrosine-protein kinase 7	3.54	2.95	PTK7	Q13308
MARK2	serine/threonine-protein kinase MARK2	3.15	2.94	MARK2	Q7KZI7
SNX8	sorting nexin-8	3.43	2.92	SNX8	Q9Y5 × 2
ACINU	apoptotic chromatin condensation inducer in the nucleus	3.18	2.87	ACIN1	Q9UKV3
2A5D	serine/threonine-protein phosphatase 2A 56 kDa regulatory subunit delta isoform	2.94	2.87	PPP2R5D	Q14738
TS101	tumor susceptibility gene 101 protein	2.67	2.83	TSG101	Q99816
SF3B1	splicing factor 3B subunit 1	3.58	2.82	SF3B1	O75533
BIRC6	baculoviral IAP repeat-containing protein 6	2.37	2.81	BIRC6	Q9NR09
PUF60	poly(U)-binding-splicing factor PUF60	2.92	2.76	PUF60	Q9UHX1
PSPC1	paraspeckle component 1	2.64	2.74	PSPC1	Q8WXF1
RBBP9	serine hydrolase RBBP9	1.80	2.62	RBBP9	O75884
STAG1	cohesin subunit SA-1	3.13	2.56	STAG1	Q8WVM7
SELO	protein adenylyltransferase SelO, mitochondrial	2.83	2.46	SELENOO	Q9BVL4
EFNMT	eEF1A lysine and N-terminal methyltransferase	3.17	2.45	METTL13	Q8N6R0
RS3A	40S ribosomal protein S3a	2.26	2.44	RPS3A	P61247
LAR4B	La-related protein 4B	2.67	2.38	LARP4B	Q92615
G3BP2	Ras GTPase-activating protein-binding protein 2	1.85	2.35	G3BP2	Q9UN86
ZO2	tight junction proteinZO-2	2.33	2.31	TJP2	Q9UDY2
RAB21	Ras-related protein Rab-21	1.75	2.27	RAB21	Q9UL25
MLEC	malectin	3.51	2.19	MLEC	Q14165
SSH3	protein phosphatase slingshot homologue 3	2.63	2.15	SSH3	Q8TE77
PEPL	periplakin	2.42	2.14	PPL	O60437
OFUT1	GDP-fucose protein *O*-fucosyltransferase 1	3.16	2.13	POFUT1	Q9H488
TOLIP	toll-interacting protein	1.72	2.11	TOLLIP	Q9H0E2
PPM1A	protein phosphatase 1A	2.25	2.06	PPM1A	P35813
SMD1	small nuclear ribonucleoprotein Sm D1	2.19	2.06	SNRPD1	P62314
PABP1	polyadenylate-binding protein 1	1.67	2.06	PABPC1	P11940
CNO11	CCR4-NOT transcription complex subunit 11	2.82	2.05	CNOT11	Q9UKZ1
KS6A1	ribosomal protein S6 kinase alpha-1	2.41	2.04	RPS6KA1	Q15418
NONO	non-POU domain-containing octamer-binding protein	2.02	2.02	NONO	Q15233
CTBP2	C-terminal-binding protein 2	2.52	2.00	CTBP2	P56545
C2D1A	coiled-coil and C2 domain-containing protein 1A	1.89	1.97	CC2D1A	Q6P1N0
SRSF7	serine/arginine-rich splicingfactor 7	2.01	1.89	SRSF7	Q16629
THTM	3-mercaptopyruvate sulfurtransferase	2.36	1.89	MPST	P25325
RL11	60S ribosomal protein L11	2.26	1.89	RPL11	P62913
KAP3	cAMP-dependent protein kinase type II-β regulatory subunit	2.04	1.88	PRKAR2B	P31323
SYFA	phenylalanine-tRNA ligase α subunit	1.84	1.85	FARSA	Q9Y285
HCFC1	host cell factor 1	2.26	1.67	HCFC1	P51610
XRCC6	X-ray repair cross-complementing protein 6	1.66	1.66	XRCC6	P12956
E41L1	band 4.1-like protein 1	1.65	1.61	EPB41L1	Q9H4G0
GDS1	Rap1 GTPase-GDP dissociationstimulator 1	–2.11	–1.55	RAP1GDS1	P52306
GPKOW	G-patch domain and KOWmotifs-containing protein	–1.58	–2.04	GPKOW	Q92917
RPAB3	DNA-directed RNA polymerases I, II, and III subunit RPABC3	–1.99	–2.51	POLR2H	P52434
PSB7	proteasome subunit βtype-7	–7.35	–3.41	PSMB7	Q99436
TYY1	transcriptional repressor protein YY1	–5.45	–3.55	YY1	P25490
TMF1	TATA element modulatory factor	–4.97	–5.66	TMF1	P82094
CSK	tyrosine-protein kinase CSK	–9.76	–6.30	CSK	P41240
DAAF9	dynein axonemal assembly factor 9	–2.71	–8.86	DNAAF9	Q5TEA3
CC170	coiled-coil domain-containing protein 170	–4.64	–9.72	CCDC170	Q8IYT3
NELFD	negative elongation factor C/D	–23.89	–36.04	NELFCD	Q8IXH7

### Other Proteins Identified in the Study

A reduced set of proteins were indicative of mild infection. Specifically, 44 proteins were identified showing differences with control and severe patients (*q* value < 0.01) (Table S3). Representative violin plots of these proteins can be found in Figure S3a. IPA revealed the presence of one major canonical pathway, the Fc epsilon signaling (Figure S3b).

We also identified a set of proteins that did not constitute real markers of an increase in the severity associated with SARS-Cov-2. These 599 proteins were found to be only significant between two of the three conditions 42 control vs mild, 301 mild vs severe, and 256 control vs severe. Notwithstanding this, a complete list and the statistical significance of each protein are shown in Table S4.

### Comparative Study

Two of the studies provided relevant information on the alterations detected in nasal mucosa 2 years after infection. First, the proteins that change exclusively in severe conditions (study 2; 228 proteins) and, second, those that changed together in mild and severe conditions vs controls (study 3, 77 proteins). Therefore, we compared them in search of similarities that shed some light on a better understanding of SARS-Cov-2 infection. Interestingly, even though the proteins of both studies were different, all the canonical pathways where these proteins were found to participate matched perfectly (Figure 5d). Especially relevant is the identification of neutrophil degranulation, Rho GTPase signaling, and autophagy as the three most important pathways.

### Upstream Regulators

Metascape analysis identified that practically 10% of the biomarkers identified in patients that suffered from severe COVID (21 proteins; labeled with # in Table 2) are CUX1 target genes (Figure S4). It is important to highlight that we have identified CUX1 itself within the protein set. Furthermore, it is remarkable that HNF4A transcription factor is predicted to act both in control and severe COVID studies by regulating the expression of 67 proteins (19 in controls and 48 in severe, IPA *p* value < 0,0005) (Table S2, upstream regulators).

## Discussion

Although the presence of biomarkers in blood in long-COVID patients two years after infection has been recently evaluated^10^ with important findings that identified members of the complement and coagulation, little is known about the presence of biomarkers that remain a few years after infection of COVID-19 once the patients have recovered from the symptoms, which could shed some light into potential mechanisms of COVID disease evolution. Taken into account that the main viral entry and therefore the first barrier against viral infection is the nasopharyngeal mucosa,^16^ we have evaluated the presence of persistent biomarkers in this tract of recovered patients two years after SARS-CoV-2 infection, considering whether they suffered mild or severe infection effects.

Previous proteomics, genomics, or transcriptomics studies that involve plasma^9,17,18^ or nasopharyngeal swabs^19−21^ of patients infected with SARS-CoV-2 have identified significant hallmarks of SARS-CoV-2 infection that allow to differentiate the severity of the symptoms of the infection both at the moment of the infection and in those patients that suffered Long-COVID-19 that turns into the observation of the differential activation of various metabolic pathways, mostly proinflammatory. A clear example is the recent study that analyzes plasma samples from COVID-19 patients that suffered long-COVID-19 after two years post infection.^10^ Although in this later study, the nature of the findings was to identify biomarkers for a more precise intervention addressed to reduce burden of long-COVID. Nonetheless, none of these studies have focused on patients who recovered two years after SARS-CoV-2 infection, showing no traces of the virus or COVID-19 symptoms while also considering the severity of their infection and whether they experienced mild or severe effects. Although we acknowledge the limitations of this study, particularly the limited number of samples for each experimental condition compared to other SARS-CoV-2-related proteomic studies and its exclusive focus on nasopharyngeal biomarkers, which may not capture systemic responses, we have successfully identified robust statistical correlations in the overall changes in protein expression across different experimental conditions.

### Mucosal Proteins that Allow to Differentiate between non-COVID, Mild, and Severe Infections

We first compared the proteomics results between the three experimental groups, non-COVID, mild, and severe. This study revealed a small group of proteins^22^ that changes among all experimental groups (Table 2 and Figure 2), which constitute the best biomarkers to differentiate between non-COVID samples and samples from patients that suffered mild and severe COVID infections. Five of these proteins, ELOC, proteasome 26S subunit non-ATPase 10 (PSMD10), ubiquitin carboxyl-terminal hydrolase 5 (UBP5), E3 ubiquitin-protein ligase (XIAP), and E3 ubiquitin-protein ligase (UBR4), are components of the ubiquitin-proteasome system (UPS), and specifically the two E3 ubiquitin ligases (XIAP and UBR4) are significantly correlated with the severity of the symptoms. Both proteins have been previously related to COVID-19 infection^22,23^ since it has been proposed that the virus utilizes host E3 ubiquitin ligases, along with its own viral proteins, to facilitate invasion, replication, escape, and inflammation, especially on epithelial cells.^24^ In the context of our study, these two proteins emerge as the most valuable biomarkers to differentiate between individuals that suffered severe and mild symptoms of COVID-19, and they also reflect that the UPS remains overstimulated at different levels two years post infection.

### Altered Proteins in the Coronavirus Infection Pathway Persist Following Severe SARS-CoV-2 Infection

The analysis of the proteins that change exclusively in severe individuals revealed that SARS-Cov-2 infection was the potential infection disease behind the observed results, and no other pathogen of any nature was found behind these results, as shown in Figure 3e. Specifically, 4 out of the 12 proteins identified to be modified in this canonical pathway (Figure 4) constitute important upstream regulators of the coronavirus pathogenesis pathway: the RNA sensor RIG-I (RIGI), eukaryotic translation initiation factor 2A (EIF2A), the apoptosis regulator BAX, and the member of the NFkB complex NFKB2. To our knowledge, neither nasopharyngeal nor plasma proteomics studies have revealed such a direct relationship with SARS-CoV-2 infection. This is especially relevant taking into consideration that the patients were infected two years before this study and that they have recovered symptoms associated with infection.

The zinc finger CCCH-type antiviral protein 1 ZCCHV (coded by gene ZC3HAV1), which is a key node in the coronavirus infection pathway as it plays an important role in the innate response against DNA and RNA viruses by binding to viral RNAs to promote their degradation and/or translation suppression, including COVID19,^25^ was also found to be increased in patients with severe disease vs non-COVID. In this line, the negative *z*-score given by IPA indicates that, overall, the coronavirus infection pathway would be somehow repressed, especially the replication steps followed by SARS-Cov-2 infection, whereas the proinflammatory response would still persist (positive *z*-score). Due to its relevance as a potential switch for virus infection, we consider this finding especially relevant.

### Other Proteins that Define Severe SARS-CoV-2 Infection

Additionally, for the proteins directly involved in the coronavirus infection pathway discussed before, we have identified a total of 228 protein markers of severe infections (vs mild and/or non-COVID samples, Table 3). Concerning inflammation, the ISG15 antiviral mechanism, which participates in the inhibition of the release and replication of viral particles, also appears to be stimulated. It has been shown that viruses from diverse families have evolved tactical strategies to manipulate ISG15-mediated antiviral responses, and perturbation of both intracellular and extracellular ISG15 functions by SARS-CoV-2 is implicated in COVID-19 hyperinflammation.^26^ In this context, several proteins associated with MHC-I function have emerged as possible key players in this intricate process and therefore may be in line with our results: the component of the inhibitor of the nuclear factor kappa B kinase complex CHUK (IKK-α) is a kinase that plays an essential role in the activation of the NF-KB pathway, thus regulating gene expression related to the immune response.^27^ Its role modulating the expression of molecules involved in antigen processing highlights its importance in the immune response to COVID-19 virus.^28^ Take into account the presence of an important number of kinases^14^ in the study, overall data reflect that inflammation is far from disappearing in SARS-CoV-2 infected patients with severe symptoms. Additional E3 ubiquitin ligases were found to be associated with severe effects of infections in our study. Thus, the deltex E3 protein (DTX3L), an E3 ubiquitin-protein ligase that associates with the ADP-ribosyltransferase PARP9, plays a role in DNA damage repair and interferon (IFN)-mediated antiviral responses.^29^ In the same context, FBXL18 and FBXO22, members of the F-box family of proteins, are associated with E3 ubiquitin-ligase complexes. These proteins regulate the stability of specific antigens by ubiquitination, labeling proteins for degradation, or modulating their activity.^30,31^ This mechanism becomes a crucial point in regulating the immune response to COVID-19. Also, as in our study, these proteins are overexpressed in certain viral infection contexts.^32^

A group of proteins involved in anterograde, retrograde, and intra-Golgi transport were also overexpressed in severe patients. Among them, we have identified several components of the COPI and COPII complexes and proteins of the clusters of orthologous groups (COG) cytosolic proteins (COG2, COG3, and COG7) with key roles in relation to retrograde transport.^15^ Other identified proteins with an important role in vesicle transport were members of the dynein/dynactin motor complex, dynactin subunits 1 (DCTN1) and 4 (DCTN4) involved in intracellular transport and NF-κB factor signaling.^33,34^ As a matter of fact, DCTN1 has been found down-regulated in human bronchial epithelial cells in COVID-19 after 24 h of infection.^35^ Proteins related to membrane stabilization and organelle organization, such as spectrins SPTAN1 and SPTBN2^36^ and USO1,^37^ were also found. In this context, RAB1B has been identified and characterized as a host factor crucial for the trafficking and maturation of the S protein of SARS-CoV-2 after its synthesis in the endoplasmic reticulum, regulating the subcellular distribution, excision, and redistribution of the S spicule after infection,^38^ which is consistent with the fact that we found greater expression of RAB1B in the group with the worst clinical course. Indeed, several proteins of the COG complex have been related to viral replication, not only for SARS-CoV-2 but also for other viruses. Recently, 15 human proteins, besides angiotensin-converting enzyme 2 (ACE2), have been identified as critical host factors for SARS-CoV-2 replication and infection, including COG3.^39^ This protein interacts with SARS-CoV-2 proteins ORF3B, ORF6, ORF7A, and ORF7B, which have also been related to inflammation and fibrotic processes,^40^ both hallmarks of patients admitted to the ICU.

Although it initially plays a protective role against infection, the sustained involvement of type I interferons over time is associated with detrimental hyperinflammation observed in severe COVID-19 patients. This is consistent with its overexpression in patients admitted to the ICU.^41^ The cytokine storm, mentioned above, with proteins of its pathway hyperactivated by SARS-CoV-2 infection, is the possible origin of severe pneumonia leading to acute lung injury, systemic inflammatory response syndrome or acute respiratory distress syndrome, multiorgan failure, or even death. It has also been described that both UBE2 V1 and the TMEM189-UBE2 V1 complex promote immune signaling, which, through elevating IL-1 levels in patients with COVID-19, originates this cytokine storm and severe inflammation, especially in severe and critical patients.^4^

SARS-CoV-2 use ACE2 as their cellular entry receptor and transmembrane serine proteinase 2 (TMPRSS2) as a priming protein for entering the host cell.^42^ Among the three receptors identified to increase with the severity of the infection, the scavenger receptor class B member 2 (SCARB2) was found to be overexpressed in mild (2,21-fold change) and severe (4,22-fold change) infections. It is interesting to indicate that this receptor has been identified as the most significant elevated protein in plasma samples of COVID-19-infected patients.^9^ Accordingly, Patel et al. also identified this receptor as an important marker of severity in COVID infection.^11^ Remarkably, studies have shown that this receptor is involved in the pathogenesis of foot and mouth disease caused by enterovirus-71, wherein it has been reported as the cellular receptor for viral infection responsible for viral entry.^43,44^ Therefore, SCARB2 is an interesting protein involved in membrane transportation and reorganization of endosomal and/or lysosomal compartments and its implication in viral entry^45^ must be further studied.

### Markers of SARS-CoV-2 Infection (Both Mild and Severe)

Further search for markers of a previous SARS-Cov-2 infection, irrespective of the severity, retrieved 77 proteins with different functions within the cell (Figure 5Table 4). Among them, 22% of the proteins^17^ were directly related to mRNA transcription or translation machinery. Specifically, processing of capped introns pre-mRNA and inhibition of ARE-mediated decay. Viral replication significantly alters the gene expression landscape of the infected cells. Many of these changes are due to viral manipulation of the host transcription or translation machinery, largely inhibiting expression by directly targeting the mRNA. In addition, several antiviral pathways use RNA degradation as a viral restriction mechanism to destroy the viral RNA.^46^ SARS-CoV-2 proteins directly engage host RNAs to dysregulate essential steps of protein production and suppress the interferon response to viral infection.^47^

In this line, targeting of the polyadenylation specificity complex (CPSF6 protein in our study) is a general mechanism of immunoevasion employed by a variety of pathogenic viruses.^48^ It is also especially relevant that two members of the SF3b multiprotein spliceosomal subcomplex (SF3B1 and SF3B4) were found overexpressed. The SF3b complex is an intrinsic component of the functional U2 small nuclear ribonucleoprotein (snRNP), facilitating spliceosome assembly and activation.^49^ The nonstructural protein NSP16 from SARS-CoV-2 binds mRNA recognition domains of U1/U2 snRNAs and disrupts mRNA splicing.^47^ Interestingly, this study also identifies 40S ribosomal protein as a key point to disrupt protein translation, a protein that was also found to increase upon viral infection.

The degradation of mRNAs at the end of their translational life, named basal decay, occurs in several steps but begins with the gradual shortening of the poly(A) tail, termed deadenylation, by cellular decay factors such as the carbon catabolite repression (CCR4-NOT) complex and poly(A)-specific ribonuclease (PARN). Deadenylation triggers the removal of the 7mG cap by the Dcp1/2 decryption complex and its activators. These events expose the RNA to rapid nucleolytic degradation by the exoribonuclease XRN1.^46^ Both CNOT11 and XRN1 were found to be overexpressed in infected patients. Therefore, our results reflect the long-lasting effect of mRNA damage as a result of COVID infection.

Interestingly, one protein that appears reduced upon infection (over 3-fold change) in patients infected by SARS-CoV-2 is the proteasome subunit beta-type 7 (PSMB7). Despite the fact that there are no direct studies of this protein in SARS infections, PSMB7 is important for capsid assembly of some viruses.^50^ Thus, the interaction between PSMB7 and the viral capsid for Grass carp reovirus protein suggests that PSMB7-mediated interference with proteasome assembly should be involved in effective virus infection.

### Markers of Mild Infections

A small group of proteins also emerged as markers of mild infections (Figure S3 and Table S3). Although the number of proteins identified in this group is reduced, activation of the Fc receptor signaling appeared as the most significant activated canonical pathway. We do not have a clear explanation of how different markers in the nasopharyngeal mucosa may allow differentiation between mild infection and control samples and severe markers of infection. Nevertheless, a recent study highlighted that a differential antibody response against SARS-CoV-2 may predict the severity of the patients.^51^ This finding supports the idea that certain antibodies against SARS-CoV-2 may exacerbate the severity of the disease via antibody-dependent enhancement, suggesting that mild and severe responses to SARS-CoV-2 may not follow the same inflammatory pathways.

### Comparative Analysis of the Results

Our final comparative analysis of the proteins that constitute hallmarks of SARS-CoV-2 infection (Table 4) with those of severe infections (Table 3) retrieves complementary results and exposes the importance of neutrophil degranulation and the Rho GTPase cycle associated with a previous infection of the virus (Figure 5d). Thus, it has been previously reported that the alteration of neutrophil degranulation processes in patients after six months of recovery from acute COVID-19 infection, even though their relationship remains uncovered.^52,53^ These patients with postacute COVID-19 syndrome had elevated levels of neutrophil extracellular traps, interpreted as a protective mechanism to kill bacteria and damage viruses.^52,53^ However, it can also affect healthy tissues, promoting inflammation being related to the development of long-term symptoms such as fatigue and shortness of breath.^54^ The serpin peptidase inhibitor SERPINB10 is the protein with the most increased levels in COVID-19 infected patients. SERPINB10 is a protease inhibitor essential for maintaining the health of the nasal cavity, producing mucus, inhibiting the exacerbated inflammation, and controlling the migration of epithelial cells and the formation of tight junctions to maintain the barrier function of the mucosal epithelium. Moreover, it has been recently reported that its up-regulation is related to inhibited apoptosis of Th2 cells.^55^ In this line, reduced apoptosis is predicted by our bioinformatics study in patients with severe effects of COVID-19 infection. Spectrin alpha, nonerythrocytic 1 (SPTAN1) is a cytoskeletal adaptor protein whose level is increased in mild/severe COVID-19, as indicated by our results. To date, no evidence has been reported to the relationship of this protein with viral infection, but some data indicates that it could be linked to inflammation and its level is inversely proportional to the infiltration of neutrophils.^56^ Hence, our results could mean that the infected cells are triggering a protective response against the virus. The Ras-related protein RAPB2, also associated with severe COVID-19 patients, is present at the membranes of gelatinase-rich granules of neutrophils, and it is speculated its role at facilitating and increasing the release of these granules.^57^ However, the gene encoding RAPB2 has been recently identified as a hotspot locus for the HPV integration into the host genome associated with cervical cancer,^58^ being tempting to speculate a similar usage by the COVID-19 virus, which is known to be integrated into the genome as reported previously.^59^ In our samples, the RNA helicase regulating RNA splicing DDX3X remains upregulated after two years from infection. DDX3X is reported to be used by COVID for its replication cycle,^60^ and also the level of the tyrdosine-protein phosphatase nonreceptor type 6, PTPN6, remained elevated in samples from mild/severe COVID infection. The role of this kinase in COVID infection is unknown, though there is evidence linking it to the genetic predisposition of COVID.^61^

The Rho GTPases function as molecular switches, cycling between an active GTP-bound state and an inactive GDP-bound state, which is tightly regulated by guanine nucleotide exchange factors (GEFs), GTPase-activating proteins, and guanine nucleotide dissociation inhibitors (GDIs). Rho proteins regulate actin polymerization to form structures related to cell morphology, cell movement, attachment, and endocytosis, being this latter process directly connected to COVID-19 infection in the virus pre-entry and endocytosis.^62^ The ephrin type-A membrane receptor 2 (EPHA2), upregulated in the mild/severe COVID-19 samples analyzed, is upstream of Rho signaling. Indeed, it has been proposed that this receptor is related to the entry of a plethora of viruses, including SARS-CoV-2, and its expression has been correlated with severe symptoms of the disease.^63^ SARS-CoV-2 spike S1 protein induces epithelial injury through the activation of the Rock2/Rho pathway, among others.^64^ Interestingly, Rho-associated coiled-coil containing protein kinase 2 (ROCK2) is a target of ephrin receptors, as EPHA2, but is also associated with the infection. GEFs are related to cytoskeletal rearrangements, cell shape, and movement. Vav guanine nucleotide exchange factor 1 (VAV1), the ABR activator of RhoGEF and GTPase (ABR), and the p21-activated protein kinase exchange factor beta (ARHGEF7) were GEFs found to be upregulated in mild/severe COVID-19 samples in our study. VAV1, added to the mentioned actions, is linked to increased levels of viral transcription and replication by triggering the JNK/SAPK signaling cascade.^65^ No data has been found for ABR and ARHGEF7, but due to their function, it would be tempting to speculate that their increase promotes cell movement necessary for wound healing as a part of the antiviral response. This hypothesis is also reinforced by the increased level of the actin scaffolding protein ACTN1, involved in cell anchorage to the substrate.^66^ In fact, this finding fits with the observed SERPINB10 level, as mentioned above.

Unravel the information regarding differential biomarkers of healthy individuals that suffered mild or severe COVID-19 symptoms would provide valuable information about a patient’s clinical history as they may help to identify those individuals that may be at higher risk for developing long-term complications, allowing also for earlier detection of potential relapses related to future COVID-19 or other viral infections. A great number of our findings are proteins related to the immune response at different levels. Therefore, understanding how these proteins change accordingly to the severity of the disease could help to design more effective vaccination strategies and more targeted treatments based on the patient’s disease history.

## Conclusions

The respiratory tract constitutes the main gate that SARS-CoV-2 uses to access the body, and therefore, it is also the most important target to investigate the mechanisms that the virus uses to initiate infection for therapeutic approaches. Herein, we prove that the effects of mild and severe SARS-CoV-2 infections remain in the nasopharyngeal mucosa two years after infection with significantly different biomarkers that define each group. These results are sustained with two main findings: the identification of potential SARS-CoV-2 infection behind the proteomic results and the correlation of the results with previous findings of metabolic alterations in infected patients. We have also exposed new proteins that may be important links to sustained invisible damage within the cells, especially for those patients that suffered severe symptoms that required ICU-level care. The finding that these biomarkers persist two years postinfection raises the question of whether the proteins identified in this study, related to RNA biosynthesis and degradation, members of the conserved oligomeric Golgi (COG) complex, or even receptors such as SCARB2, or EPHA2 are modified as a consequence of SARS-CoV-2 infection, or whether previously reduced or increased constitutive levels of these proteins in infected individuals played a determinant role in their severity of response to the infection.

## Data Availability

All the additional data that was part of the current study is included as Supporting Information.

## Supporting Information Available

Raw data obtained from directly from DIA-PADEF proteomics study including *p* and *q* values (XLSX)
Components of all the proteins that participate in canonical pathways using IPA in all the comparative conditions (XLSX)
Description of the 44 proteins that were found differentially expressed in mild group vs the other groups (*q* < 0.01) proteins (XLSX)
Description of the 599 proteins that were found differentially expressed only in one vs another condition (*q* < 0.01) proteins (XLSX)
Sample reproducibility, reproducibility of replicate measurements is expressed in three different analyses, protein biomarkers characteristics of patients with mild effects of COVID-19 infections compared to the other two groups, and upstream regulators of the proteins that change in severe COVID infections vs the rest of the groups identified using Metascape and IPA (PDF)
Sample reproducibility, reproducibility of replicate measurements is expressed in three different analyses, protein biomarkers characteristics of patients with mild effects of COVID-19 infections compared to the other two groups, upstream regulators of the proteins that change in severe COVID infections vs the rest of the groups identified using Metascape and IPA, raw data obtained from directly from DIA-PADEF proteomics study including p and q values. Components of all the proteins that participate in canonical pathways using IPA in all the comparative conditions, description of the 44 proteins that were found differentially expressed in the mild group vs the other groups (*q* < 0.01) proteins, and description of the 599 proteins that were found differentially expressed only in one vs another condition (*q* < 0.01) proteins (PDF)
