# Supplementary material for: Longitudinal Assessment of Nasopharyngeal Biomarkers Post-COVID-19: Unveiling Persistent Markers and Severity Correlations
Source: J Proteome Res. 2024 Oct 11;23(11):5064–84. doi: 10.1021/acs.jproteome.4c00536 (PMC11536464; doi:10.1021/acs.jproteome.4c00536)
Supplement: Supplementary file 6 — pr4c00536_si_006.pdf [file pr4c00536_si_006.pdf]

Supporting information for publication

Longitudinal Assessment of Nasopharyngeal Biomarkers Post-COVID-19: Unveiling Persistent Markers and Severity Correlations

Redondo-Calvo Francisco Javier[1,2,3]*, Rabanal-Ruiz Yoana[4,7]*, Verdugo-Moreno Gema[2], Bejarano-Ramírez Natalia[2,3,5], Bodoque-Villar Raquel[2], Durán-Prado Mario[4,7], Illescas Soledad[6], Chicano-Galvez Eduardo[8], Gómez-Romero Francisco Javier[2], Martinez-Alarcón José[6], Arias-Pardilla Javier[2], Lopez-Juarez Pilar[2], Padin Juan Fernando[4,7], Peinado Juan Ramón[4,7#], Serrano-Oviedo Leticia[2#].

*Both authors contributed equally
#Corresponding authors

[1]Department of Anesthesiology and Critical Care Medicine, University General Hospital, 13004 Ciudad Real, Spain, SESCAM.
[2] Traslational Investigation Unit, University General Hospital, Ciudad Real, Spain. SESCAM. Research Institute of Castilla-La Mancha (IDISCAM).
[3]Faculty of Medicine, University of Castilla-La Mancha, Castilla La Mancha, Ciudad Real, Spain
[4]Oxidative Stress and Neurodegeneration Group, Medical Sciences Department, Medical School, UCLM, Ciudad Real, Spain. Regional Centre for Biomedical Research, University of Castilla-La Mancha, Ciudad Real, Spain. Instituto de Investigación Sanitaria de Castilla-La Mancha (IDISCAM), Toledo, Spain.
[5]Department of Pediatrics, University General Hospital, 13004 Ciudad Real, Spain.
[6]Department of Microbiology, University General Hospital, 13004 Ciudad Real, Spain.
[7]Department of Medical Sciences, School of Medicine at Ciudad Real, University of Castilla-La Mancha, Ciudad Real, Spain.
[8]IMIBIC Mass Spectrometry and Molecular Imaging Unit (IMSMI). Maimonides Biomedical Research Institute of Cordoba (IMIBIC), Reina Sofia University Hospital, University of Cordoba (UCO), 14004 Córdoba, Spain.

Suppl. Fig 1. Samples reproducibility. The analysis of chromatography data for the internal quality control (QC) samples demonstrates good reproducibility between runs and confirms the stability of the chromatography across sample acquisitions. (a) Base Peak Chromatograms for all internal QC samples using the 60SPD Evosep method. The accuracy of the retention time (RT) prediction during the chromatographic separation step was evaluated as an indicator of experiment performance, using the Spectronaut results. The median absolute delta RT accuracy calculated across all samples analyzed was 0.02 min in 21 minutes of total chromatographic time. (b) Some randomized samples were analyzed in duplicate during the acquisition to ensure sample reproducibility. Identified and quantified proteins in this sample QC were compared by correlation analysis.

Suppl. Fig 2. The reproducibility of replicate measurements is expressed in three different analyses. First, the difference between peptide intensities in each sample are compared to the mean value among all replicates (a). Next, the Coefficient of Variation (CoV) is used as a metric

for reproducibility to explore how much the CoV within a sample group can be improved by removing a single sample (b). Finally, the Coefficient of Variation (CoV) can be visualized using box- and violin-plots, either at peptide or protein levels (c and d).

Suppl. Fig 3. Protein biomarkers characteristics of Patients with mild effect of COVID-19 infections compared to the other two groups ($q<0.01$). a) Violin-plot of representative proteins that display increased expression (top) and decreased expression in this experimental group (bottom). b) IPA analysis of the canonical pathways.

Suppl. Fig 4. Upstream regulators of the proteins that change in severe covid infections vs the rest of the groups identified using Metascape (a) and IPA (b). CUX1 and EGFR are highlighted as the most significant findings. Proteins controlled by Cux1 are indicated with # in Table 3.

Suppl. table 1. Raw data obtained from directly from DIA-PADEF proteomics study including p and q values.
Suppl. table 2. Components of all the proteins that participate in canonical Pathways using IPA in all the comparative conditions.
Suppl. table 3. Description of the 44 proteins that were found differentially expressed in mild group vs the other groups ($q<0.01$) proteins). Intensities and Fold change are indicated.
Suppl. table 4. Description of the 599 proteins that were found differentially expressed only in one vs another condition ($q<0.01$) proteins). Intensities and Fold change are indicated.
